# Diagnostic challenges in hepatic metastatic olfactory neuroblastoma: a case report and literature review

**DOI:** 10.1007/s12672-026-04689-8

**Published:** 2026-03-29

**Authors:** Ke He, Dongyue Wen, Jinshu Pang, Hong Yang

**Affiliations:** Department of Medical Ultrasound, First Affiliated Hospital of Guangxi Medical University, Nanning, China

**Keywords:** Olfactory neuroblastoma, Hepatic metastasis, Primary hepatocellular carcinoma, Imaging misdiagnosis, Hepatic imaging

## Abstract

**Background:**

Olfactory neuroblastoma (ONB) is a rare malignant tumor arising from the nasal cavity and paranasal sinuses, often presenting diagnostic challenges due to its potential for recurrence and distant metastasis, particularly to uncommon sites such as the liver.

**Case presentation:**

We report the case of a 42-year-old female with a history of ONB who was admitted after a liver mass was detected during routine examinations at another hospital two months earlier. The lesion was initially misdiagnosed as primary hepatocellular carcinoma based on imaging findings from contrast-enhanced ultrasound, enhanced CT, and MRI, which revealed a focal lesion in liver segments S5/6. Laboratory results showed slightly elevated AFP (8.91 ng/ml) and CA19-9 (37.12 U/ml), with no other significant abnormalities. The patient subsequently underwent open liver resection.

**Results:**

Postoperative pathological examination revealed a malignant small round blue cell tumor. Histomorphological and immunohistochemical findings, in conjunction with the patient’s clinical history, confirmed the diagnosis of hepatic metastasis from ONB.

**Conclusion:**

Hepatic metastasis of ONB can closely mimic primary liver cancer on imaging, underscoring the necessity of integrating comprehensive clinical history, imaging assessment, and pathological confirmation to avoid misdiagnosis.

## Introduction

Olfactory neuroblastoma (ONB) is a rare malignant tumor originating from the primitive basal cells of the olfactory neuroepithelium and belongs to the neuroendocrine tumor family [1]. ONB has a moderate tendency to metastasize (10–30%), with cervical lymph nodes being the most common site, while hepatic metastasis being rare [2–4]. The clinical and imaging manifestations of hepatic metastatic ONB lack specificity, often leading to misdiagnosis or missed diagnosis in clinical practice [5]. The rarity of hepatic metastasis and its non-specific imaging features often lead to misdiagnosis as primary liver cancer or other hepatic malignancies. This case report discusses the diagnostic challenges encountered in a patient with hepatic metastatic ONB, summarizes the imaging characteristics and reasons for the misdiagnosis, and suggests diagnostic approaches to improve the understanding of this disease among clinical and radiological physicians, with the aim of enhancing early diagnostic capabilities.

## Case presentation

A 42-year-old female was admitted for evaluation of a liver mass detected during routine examinations at another hospital two months earlier. The patient has a history of ONB, which was treated with surgical resection and radiotherapy. The tumor was classified as Hyams grade 2, staged as Kadish C, and had a TNM staging of T2N0M0. Initially, it involved the left nasal cavity, extending into the posterior ethmoid sinuses and sphenoid sinuses, but with no evidence of intracranial extension on imaging. Regular follow-up scans have shown no recurrence of the disease.

At present, the patient was asymptomatic, with no complaints of nausea, vomiting, chest tightness, shortness of breath, or fever. There was no history of hepatitis, relevant family history, or known genetic disorders. Physical examination was unremarkable. Laboratory tests revealed slightly elevated AFP (8.91 ng/ml, normal range: <7 ng/ml) and CA 19 − 9 (37.12 U/ml, normal range: <37 U/ml), with other tumor markers (CEA, CA125, CA153, PIVKA-II) within normal limits.

**Fig. 1:**
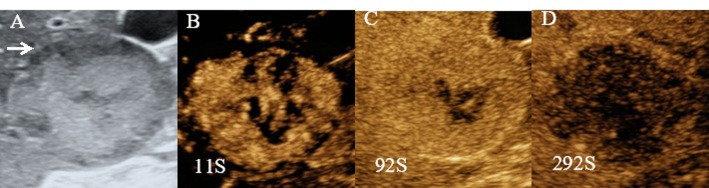
Contrast-enhanced ultrasound of liver segments V and VI. **A** Routine ultrasound showing a slightly hypoechoic lesion. **B** Arterial phase peripheral hyperenhancement mimicking HCC. **C**, **D** Portal and delayed phases showing progressive washout, leading to an initial diagnosis of HCC

**Fig. 2:**
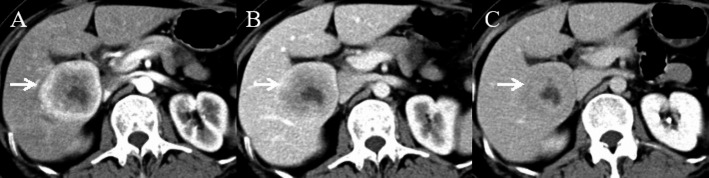
Contrast-enhanced CT of the lesion. **A** Arterial phase ring-like enhancement resembling massive HCC. **B** Portal venous phase with reduced enhancement. **C** Delayed phase with central fill-in and non-enhancing areas, reinforcing suspicion of primary liver cancer

**Fig. 3:**
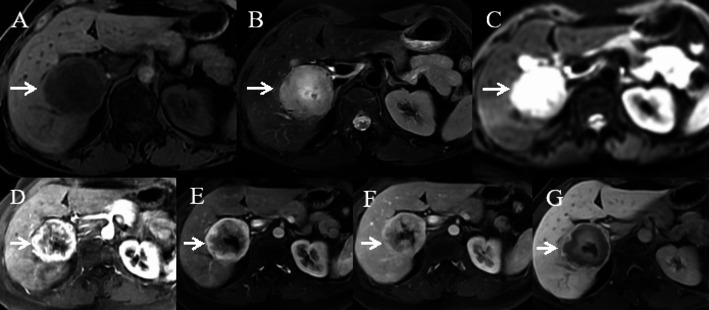
Gd-EOB-DTPA MRI of the lesion. **A**, **B** Long T1 and mildly prolonged T2 signals. **C** Restricted diffusion on DWI. **D**,** E** Arterial and portal phases showing heterogeneous ring enhancement and partial washout, suggestive of HCC. **F** Delayed phase central enhancement. **G** Low hepatobiliary phase signal, consistent with initial misdiagnosis

Imaging studies, including contrast-enhanced ultrasound, CT, and Gd-EOB-DTPA MRI, demonstrated a 6.1 × 4.8 cm mass in liver segments S5/6, with clear margins. The mass showed heterogeneous ring-like enhancement in the arterial phase, with reduced enhancement in the portal and delayed phases, suggesting the possibility of primary hepatocellular carcinoma (HCC). On contrast-enhanced ultrasound (Fig. 1), peripheral hyperenhancement during the arterial phase was noted, which initially led to the misdiagnosis of HCC. Enhanced CT (Fig. 2) and MRI (Fig. 3) showed similar characteristics, further supporting the diagnosis of HCC.

**Fig. 4:**
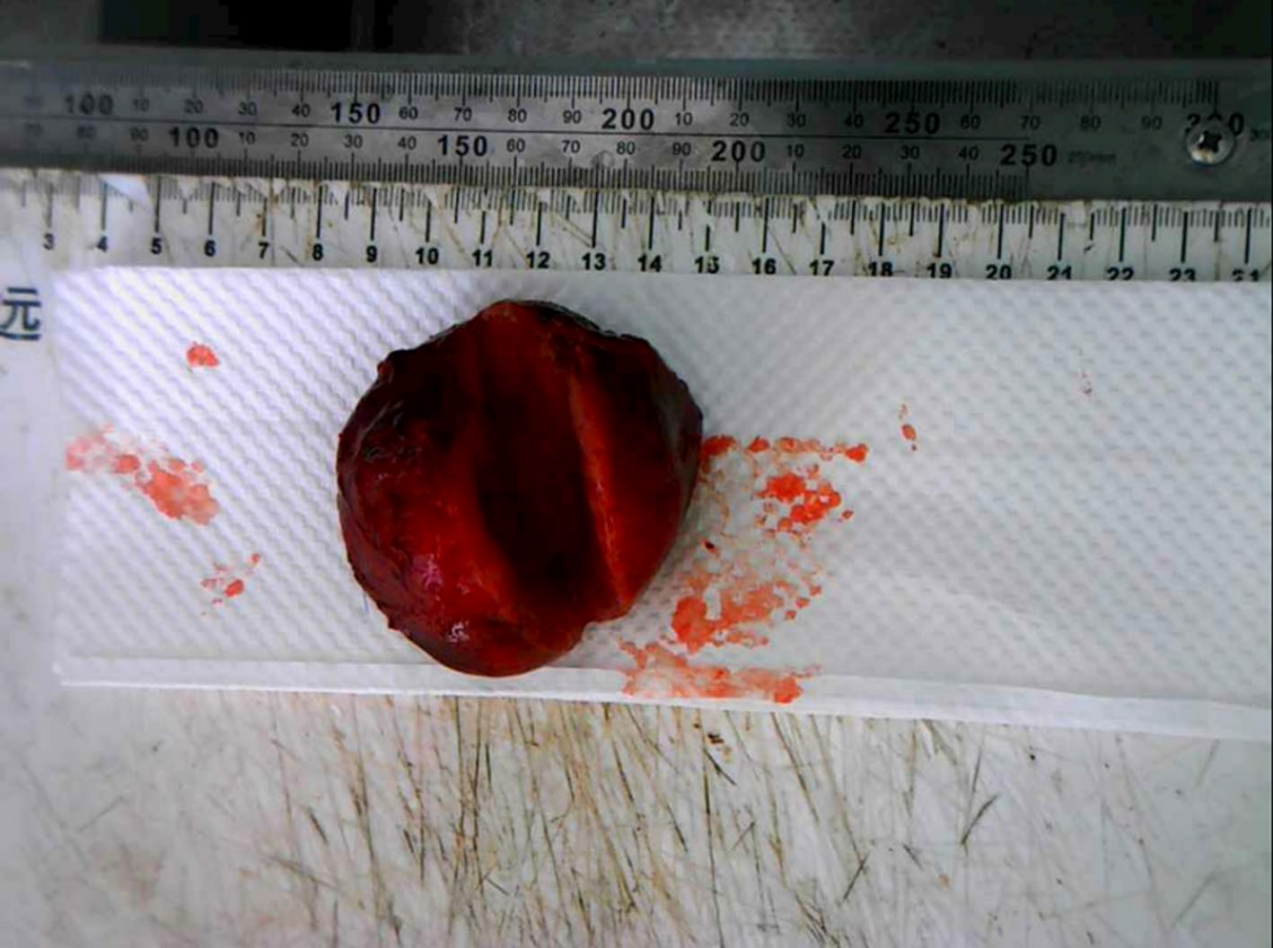
Gross pathology of the resected lesion. Gray-white to gray-yellow solid mass, 6 × 6 × 4 cm, with firm texture and capsule—features not specific to HCC

**Fig. 5:**
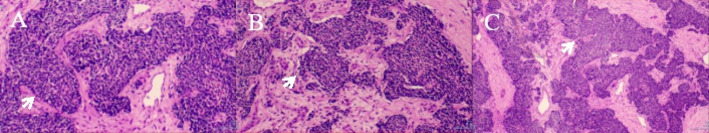
Microscopic pathology of the lesion. **A**–**C** Small round blue cell morphology. Immunohistochemistry confirmed metastatic ONB (CgA+, Syn+, CD56+), explaining the misdiagnosis based on imaging alone

The patient underwent open liver resection. Postoperative gross pathology (Fig. 4) revealed a well-circumscribed mass with a gray-white to gray-yellow cut surface. Microscopic examination (Fig. 5) showed a malignant small blue cell tumor. Immunohistochemistry demonstrated positivity for chromogranin A (CgA), synaptophysin (Syn), and CD56, with a Ki-67 labeling index of approximately 30%, consistent with hepatic metastatic ONB, in light of the patient’s clinical history.

## Discussion

ONB is a rare tumor that originates from the olfactory nerve or its associated tissues [6]. It typically presents as a localized mass with invasion [7], and has the potential for distant metastasis. The metastasis rate of ONB ranges from 10% to 30%, with cervical lymph nodes being the most common site [8]. Other metastatic sites include the lungs, pleura, central nervous system, and bones, while liver metastasis is relatively rare [9, 10].

Research on ONB has primarily focused on its pathogenesis and treatment methods [3, 11], with limited studies on its radiological features, especially in liver metastasis. Liver metastasis from ONB can mimic primary liver cancer, cholangiocarcinoma, and other malignant liver tumors due to its non-specific imaging characteristics.

The main reasons for the misdiagnosis in this case include:

- ➀ Overlapping Imaging Features: Hepatic metastases from ONB may resemble hepatocellular carcinoma (HCC), showing arterial phase hyperenhancement and portal venous phase washout, typical of HCC [12]. However, ONB metastasis may also exhibit delayed washout, further mimicking HCC, as seen in this case (Figs. 1, 2 and 3). Similar patterns were observed on enhanced CT and MRI, leading to misdiagnosis [13, 14].
- ➁ Non-Specific Laboratory Findings: Tumor markers like AFP and CA19-9, which are elevated in primary liver cancer [15] and cholangiocarcinoma [16], may also be mildly elevated in liver metastases from ONB [17]. Although ONBs typically do not secrete AFP or CA19-9 [11], rare cases can lead to mild elevations, confusing the diagnosis and suggesting mixed-type liver cancer or other liver malignancies [18–21].
- ➂ Clinical Context Oversight: Despite the patient’s history of ONB, the low incidence of liver metastasis may have led clinicians to overlook this possibility. Early liver metastasis often lacks characteristic symptoms, which reduces clinical suspicion [22].
- ➃ Limited Experience with Rare Diseases: ONB and its hepatic metastasis are rare, and many clinicians may lack experience in recognizing these conditions. The similarity in imaging to more common liver cancers, like HCC, increases the likelihood of misdiagnosis [23, 24].
- ➄ Lack of Preoperative Biopsy: The absence of a preoperative liver biopsy was a critical factor in the misdiagnosis. A biopsy would have provided definitive evidence of metastatic disease, preventing the mistaken diagnosis of primary liver cancer. Preoperative biopsy is vital when imaging and laboratory findings are inconclusive [25–27].

Therefore, the misdiagnosis of hepatic metastatic ONB as primary liver cancer results from a combination of overlapping imaging features, non-specific laboratory findings, clinical context oversight, limited experience with rare diseases, and the lack of preoperative biopsy. To improve diagnostic accuracy, a comprehensive approach integrating imaging, laboratory results, and clinical history, along with preoperative biopsy, is essential [28, 29]. Further research and education on rare metastases, particularly from ONB, are needed to reduce misdiagnosis rates [30].

## Conclusion

Hepatic metastatic ONB can closely mimic primary liver cancer on imaging, leading to misdiagnosis. To improve diagnostic accuracy, clinicians should integrate the patient’s clinical history, perform preoperative biopsy when feasible, and correlate imaging and laboratory findings with histopathological confirmation. A comprehensive approach, including early suspicion of rare metastases and consideration of a patient’s oncological history, is essential to reduce misdiagnosis rates and ensure timely, appropriate treatment.

## Data Availability

Data availability All relevant data supporting the findings of this case report are included within the manuscript. No additional datasets were generated or analyzed.
